# Plankton response to global warming is characterized by non-uniform shifts in assemblage composition since the last ice age

**DOI:** 10.1038/s41559-022-01888-8

**Published:** 2022-10-10

**Authors:** Tonke Strack, Lukas Jonkers, Marina C. Rillo, Helmut Hillebrand, Michal Kucera

**Affiliations:** 1 MARUM - Center for Marine Environmental Sciences, University of Bremen, Bremen, Germany; 2 Institute for Chemistry and Biology of the Marine Environments (ICBM), University of Oldenburg, Wilhelmshaven, Germany; 3 Helmholtz Institute for Functional Marine Biodiversity at the University of Oldenburg (HIFMB), Oldenburg, Germany; 4 Alfred Wegener Institute (AWI), Helmholtz-Centre for Polar and Marine Research, Bremerhaven, Germany

**Keywords:** Biogeography, Biodiversity, Macroecology, Palaeoecology, Community ecology

## Abstract

Biodiversity is expected to change in response to future global warming. However, it is difficult to predict how species will track the ongoing climate change. Here we use the fossil record of planktonic foraminifera to assess how biodiversity responded to climate change with a magnitude comparable to future anthropogenic warming. We compiled time series of planktonic foraminifera assemblages, covering the time from the last ice age across the deglaciation to the current warm period. Planktonic foraminifera assemblages shifted immediately when temperature began to rise at the end of the last ice age and continued to change until approximately 5,000 years ago, even though global temperature remained relatively stable during the last 11,000 years. The biotic response was largest in the mid latitudes and dominated by range expansion, which resulted in the emergence of new assemblages without analogues in the glacial ocean. Our results indicate that the plankton response to global warming was spatially heterogeneous and did not track temperature change uniformly over the past 24,000 years. Climate change led to the establishment of new assemblages and possibly new ecological interactions, which suggests that current anthropogenic warming may lead to new, different plankton community composition.

## Abstract (web summary)

Analysing a compilation of planktonic foraminifera assemblage time series covering the past 24,000 years, from the last ice age to the current warm period, the authors find that responses to warming were highly heterogeneous leading to the emergence of novel assemblages and possibly new ecological interactions.

## Main

Climate change affects biodiversity on multiple time scales. On longer time scales, species may adapt or go extinct. On shorter time scales, climate change will first affect species biogeography because in the absence of physical barriers, species can respond to change by habitat tracking—a central concept in global change ecology^1,2^. Range shifts in response to the ongoing global warming have been documented in many species across ecosystems (for reviews, see refs. ^3–5^), but because of the lack of barriers and high dispersal potential due to currents, habitat tracking should be particularly widespread in marine plankton^6–8^. Although habitat tracking may be induced by a single forcing factor, the migrating species will experience novel direct and indirect ecological interactions with other species that did not occur in their original habitat. Therefore, range shifts driven by changes in abiotic conditions are probably modified by ecological complexity, such as the emergence of new ecological interactions^9^. Moreover, the ecological niche of a species depends on multiple abiotic parameters, which may not all change at the same pace across space. Therefore, range shifts in response to environmental change may differ among species and proceed at different paces, resulting in the establishment of novel communities that differ from those existing before the environmental change. There is indeed evidence for such novelty as we observe asymmetry between the leading and trailing edge of ongoing species expansions^7,10–12^, which creates new assemblages composed of expanding species meeting persisting ones. Besides the effects on biodiversity and species richness, asymmetrical range shifts and the resulting novel ecological interactions may also have important consequences for the overall functioning of ecosystems, including effects on trophic interactions, material flow, primary production and biogeochemical cycles^13–16^.

Biological monitoring of biodiversity change can inform us about current patterns^7,10^ and rates^7,17^ of species response to environmental change. However, such monitoring cannot fully encompass the long-term ecological outcomes of environmental change because it rarely spans more than a century^10,18^ and the magnitude of environmental change in many key parameters over the monitored period is small compared with the probable extent of future global change. In many parts of the ocean, however, sedimentary microfossil records of hard-bodied plankton groups are available, with resolution sufficient to study biodiversity change across millennia, covering larger magnitudes of environmental change (for example, the warming associated with the transition from the last ice age to the current warm period^19^). Although the majority of plankton biomass is composed of soft-bodied groups that are not preserved in the fossil record^20^, the diversity of marine microfossils correlates globally with overall marine diversity^21^. Plankton groups with fossil records can therefore serve as a proxy to study plankton biodiversity change in the past and inform us about what to expect in the future. However, their potential to reveal the ecological changes of the planktonic communities on a basin-wide scale during the last climatic upheaval has never been exploited.

One of the most complete microfossil records among marine plankton is that of planktonic foraminifera^22^—calcifying zooplankton that inhabit the upper water layer of all ocean basins. They interact with other plankton groups through photosymbiosis^23^, predation or grazing^24^. Their spatial distribution and species turnover are sensitive to sea-surface temperature^8,25^, resulting in a strong latitudinal diversity gradient (LDG)^26–30^ and a detectable response to the ongoing global warming^31^, which has also been documented in a range of other marine plankton groups^17,32–34^. Owing to their excellent fossil record, resolved and standardized taxonomy, and the existence of large datasets initially generated to reconstruct past climate^35–38^, the fossil record of planktonic foraminifera has been widely used to investigate long-term changes in marine plankton biodiversity^39–41^ and biogeographic patterns^29,30^. Since there is no evidence for extinctions or the emergence of new species of planktonic foraminifera in the late Quaternary^42^ and the thermal niche of the extant species is considered to have been stable over the last glacial cycle^43^, planktonic foraminifera should have responded to the rapid temperature rise that accompanied the end of the last ice age by habitat tracking, resulting in an immediate and directional response. If planktonic foraminifera species responded predominantly by habitat tracking, the assemblage compositional change should be scaled to the environmental forcing, resulting in the conservation of assemblage composition, which would have shifted in pace with the movement of the constituent species. However, if the biotic response involved processes beyond temperature-driven habitat tracking, the fossil record should reveal an ecological response that was not always in pace with the environmental forcing, and potentially the emergence of novel assemblages. Distinguishing between these possible trajectories is important to assess the long-term response of plankton biodiversity to global change.

**Fig. 1:**
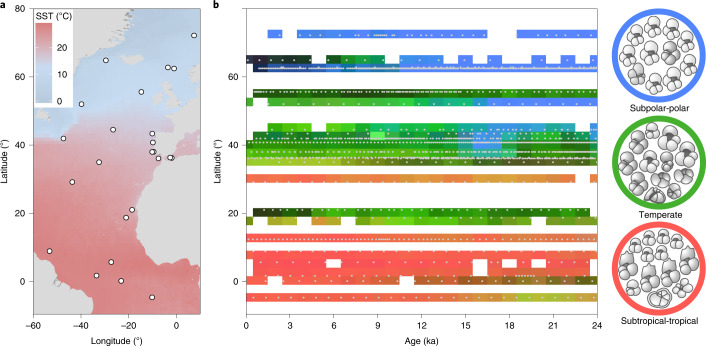
Transformation of planktonic foraminifera assemblage composition in the North Atlantic since the last ice age. **a**, Location of 25 analysed planktonic foraminifera assemblage time series (white circles). Background: modern annual mean sea-surface temperature (SST) from the WOA18^79^. **b**, Visualization of the spatio-temporal pattern of the overall assemblage change, with the first three PC of dissimilarity serving as RGB coordinates (see Methods) for each analysed assemblage (grey dots), gridded at 1 kyr by 2.5° latitude. Similar colours in the grid correspond to similar species compositions. The three circles on the right side show exemplary compositions of the three main assemblages visualized in **b**. We are aware that the RGB colour palette is not colour-blind friendly and provide another version of **b** in Extended Data Fig. 1.

Here we compile a coherent spatio-temporal dataset of 25 time series of planktonic foraminifera assemblage (sensu ref. ^44^) composition that are distributed along the full latitudinal gradient of the North Atlantic Ocean and span the past 24 thousand years (kyr) with an average resolution of 600 years (Fig. 1a and Extended Data Table 1). The time series cover the time from the last ice age across the deglaciation to the present warm period, spanning a climatic upheaval with a magnitude comparable to the probable extent of future global warming^45^. We use global mean surface temperature as a measure of climate change and analyse time series of biodiversity change to explore how the past environmental change related to the observed species redistributions and changes in assemblage composition through time. Our analyses reveal immediate and directional shifts in the distribution of assemblages during the temperature rise that accompanied the end of the last ice age, but a large component of the change in assemblage composition post-dates the rapid deglacial warming and we detect the emergence of novel assemblages during the climatically rather stable current warm period. Remarkably, the rate of community change during the current warm period is as high as during the deglaciation, even though the environmental forcing by global temperature is much weaker.

## Results

**Extended Data Fig. 1:**
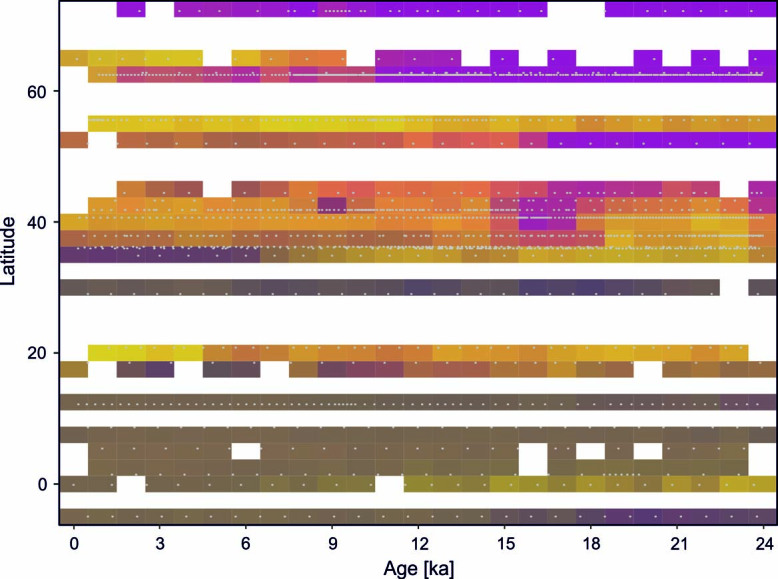
Colour-blind friendly visualisation of the spatio-temporal pattern of the overall assemblage change. This figure shows the colour-blind friendly version of Fig. 1b for each analysed assemblage (grey dots), gridded at 1 ka by 2.5° latitude. We again use the first three PCs that explain more than 97 % of the variance in the compositional dissimilarity matrix (see method section), but instead of assigning RGB values to the PC scores, we here mix different colour palettes for each PC using individual colours from the plasma colour palette. One colour palette was calculated for PC1 (from yellow to purple) and another one for PC2 (from orange to dark blue). These palettes were then mixed with the ratio defined by PC3. Similar colours in the grid correspond to similar species compositions.

We analysed 25 time series of planktonic foraminifera abundance data across the latitudinal gradient of the North Atlantic Ocean (Fig. 1a and Extended Data Table 1). The species composition of all samples of this dataset indicated the presence of three main assemblages: subpolar-polar, temperate and subtropical-tropical (Fig. 1b). Across the past 24 kyr, there was a systematic transformation of assemblage composition from colder towards warmer species compositions (Fig. 1b and Extended Data Fig. 1). The largest transformation occurred in the mid latitudes, where subpolar-polar assemblages were replaced by temperate ones over the transition from the last ice age to the current warm period. With the beginning of the current warm period (at around 11 thousand years ago (ka)), subtropical-tropical assemblages expanded poleward, south and north of the equator (Fig. 1b). At around 6–9 ka, temperate species migrated poleward to about 65° N. In the mid latitudes, the prevalence of temperate assemblages was interrupted by a transient emergence of subpolar-polar assemblages at 15–17 ka, associated with a well-known cold period (Heinrich Event) with icebergs reaching south to the Iberian Margin^46–48^.

**Fig. 2:**
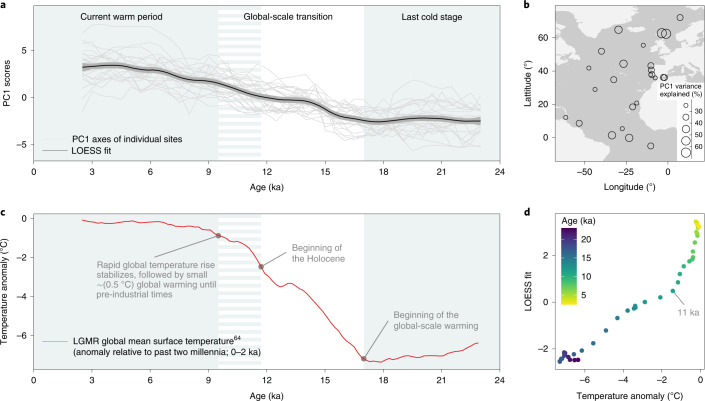
Planktonic foraminifera assemblage response to global warming during the past 24 kyr. **a**, Compositional change within individual time series shown as first principal component (PC1) axes scores (grey lines, interpolated at 0.5 kyr) and overall compositional change shown as a locally fitted polynomial regression line (LOESS fit, black line) and its 95% confidence interval (grey shading). **b**, Variance explained by individual PC1 axes at each site. **c**, Development of global mean surface temperature^64^ (red line). The temperature anomaly is referenced to the past two millennia (0–2 ka). **d**, Comparison of overall compositional change (LOESS fit) and global warming (temperature anomaly). LGMR, last glacial maximum reanalysis.

The principal component (PC) of assemblage change suggests a unidirectional transformation (Fig. 2a), with the first PCs of the individual faunal trends explaining 20.4–65.3% of the variance in each time series (Fig. 2b). Initially, the assemblage composition tracked the global temperature forcing from the last ice age until around 11 ka (Fig. 2a,c). Then, assemblage change seemed to decouple from temperature, as the faunal change continued at the same pace for about 6 kyr despite a much smaller magnitude of warming during this time (Fig. 2d).

**Fig. 3:**
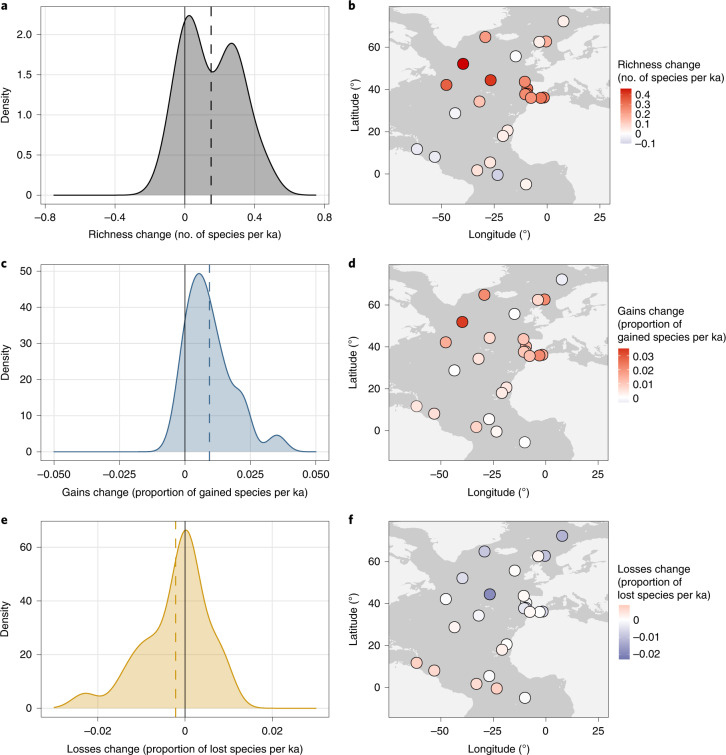
Local rates of biodiversity change of planktonic foraminifera in the past 24 kyr. **a**–**f**, Probability density functions and spatial distributions of rates of change in species richness (**a**,**b**) and the proportion of gained (**c**,**d**) and lost (**e**,**f**) species since the LGM. The rate of change is quantified for every time series as the slope of fitted linear models (see Methods and Extended Data Figs. 3 and 4). Dashed vertical lines in the probability density functions indicate the overall mean in richness (**a**), gains (**c**) and losses (**e**), and solid black lines indicate zero. Note the different scales of individual plots.

**Fig. 4:**
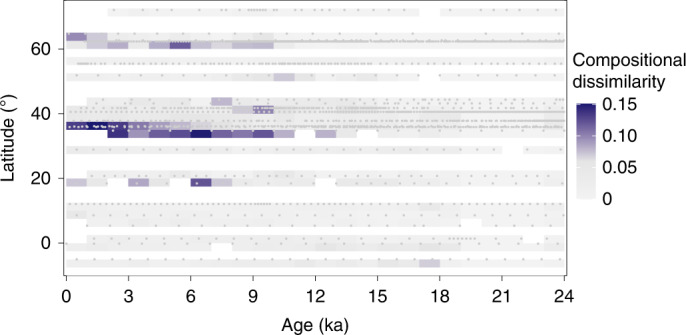
The emergence of no-analogue assemblages of planktonic foraminifera in the North Atlantic since the LGM. No-analogue assemblages are identified by the compositional dissimilarity (Morisita-Horn distance) between a sample (grey dots) and the nearest LGM (19–23 ka) analogue being higher than 0.06 (99th percentile of nearest-analogue distances within the LGM dataset; Extended Data Fig. 5). Grid cells with values above and below the 0.06 threshold value are coloured in purple and grey, respectively. Grid cell resolution of the visualization: 1 kyr by 2.5° latitude.

Over the past 24 kyr, the largest changes in species richness occurred in the mid latitudes and richness in the tropics remained unchanged (Fig. 3a,b). The gains and losses components of the species richness change reveal an asymmetry between local colonizations and extinctions, with the magnitude of local colonization outpacing local extinctions (Fig. 3c–f). The overall accumulation of species gains (Fig. 3c) can be attributed to the mid latitudes where species gains were highest (Fig. 3d). In contrast, species losses were greatest in the tropics since the last deglaciation but neutral or lower in the mid and high latitudes (Fig. 3f), with an overall lower magnitude (Fig. 3e). The poleward migration of planktonic foraminifera species into new environments (Fig. 1b) and the persistence of the original species in these same areas (Fig. 3) led to the formation of new mid-latitude assemblages without analogues in the glacial ocean (Fig. 4). With the beginning of the current warm period, these mid-latitude assemblages became compositionally even more dissimilar from assemblages that were present during the Last Glacial Maximum (LGM; 19–23 ka). Progressively, the composition of assemblages at higher (around 60° N) and lower (around 20° N) latitudes also departed from their nearest LGM analogues (Fig. 4).

**Fig. 5:**
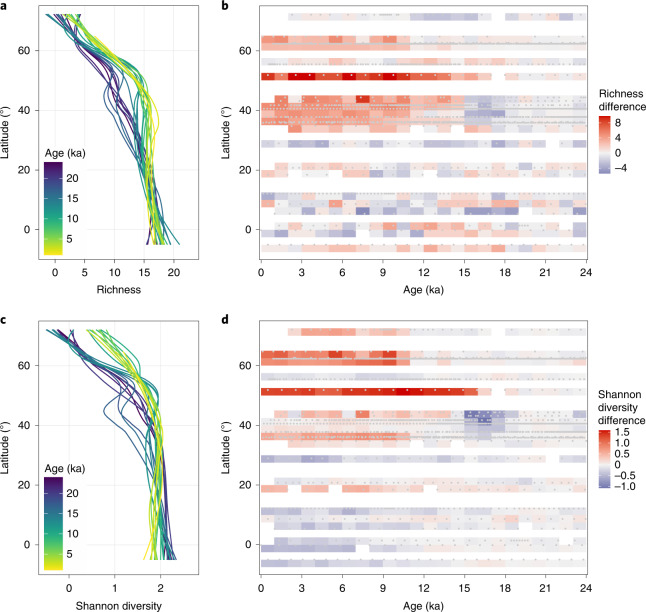
Evolution of the LDG in North Atlantic planktonic foraminifera for the past 24 kyr. **a**,**c**, LDGs based on species richness (**a**) and Shannon diversity (**c**) for the past 24 kyr expressed as locally fitted polynomial regression lines (LOESS fit) for all samples falling within one millennium. **b**,**d**, Differences in species richness (**b**) and Shannon diversity (**d**) from the LGM mean of each time series gridded at 1 kyr by 2.5° latitude. Grey dots represent individual samples.

The asymmetry of local immigration and local extinction and the resulting transformation of the assemblage composition since the last ice age affected the development of the planktonic foraminifera LDG in the North Atlantic Ocean (Fig. 5). The shape of the LDG continuously changed throughout the past 24 kyr. The largest transformation of the LDG occurred between 30 and 50° N, with an initial transient decrease in species richness (Fig. 5a) and Shannon diversity (Fig. 5c) between 15 and 17 ka, followed by a steady increase with highest values in the most recent time slices. At high latitudes, Shannon diversity and species richness remained stable over the transition from the last ice age to the current warm period but increased at around 11 ka, with the increase in diversity being more prominent. Although the number of species in the tropics remained relatively stable during the past 24 kyr (Fig. 5b), Shannon diversity progressively declined (Fig. 5d), leading to the flattening of the LDG in the tropics and ultimately the development of the present-day tropical diversity dip (Fig. 5c).

## Discussion

Even though the rate of global warming has markedly reduced with the beginning of the current warm period when compared with the last deglaciation, our observations indicate that planktonic foraminifera assemblages continued to change at the same pace as during the deglaciation after the transition to the current warm period ended (Fig. 2a,c). This continuous transformation of assemblages during the current warm period lasted for at least 6 kyr after the temperature forcing had stabilized (Fig. 2), and could reflect the restructuring of ecological interactions, responses to other changing abiotic variables, and/or neutral drift^49,50^. However, if neutral drift were the main control on turnover, one would expect assemblage change to be out of pace with climate change during the deglaciation and also to occur during the climatically stable period at the end of the last ice age before the onset of global warming (before 17 ka), which is not the case (Fig. 2a). Alternatively, the continued assemblage change could reflect community restructuring due to asymmetric gains and losses during the warming-forced assemblage transformation (Fig. 3). Prolonged phases of imbalance between local immigration and extinction have indeed been proposed for several species groups^51–53^. This mechanism would imply that the timescale for reaching a new equilibrium in species turnover could be longer than the elapsed current warm period, indicating a very long (>10 kyr) lag between temperature forcing and plankton response.

While assemblage turnover can take centuries to millennia to stabilize, as shown for many tree species and large mammals^53^, our data show evidence against a lag in the response that is longer than the century-scale resolution of our time series. First, we observe no changes in the assemblage composition during the climatically stable period between 17 and 23 ka before the onset of global warming (Fig. 2), even though this period was directly preceded by rapid and pronounced climate change before 27 ka^54^. If there was a very long lag between forcing and plankton response, we would also expect to see an influence between 17 and 23 ka. Second, the local prevalence of subpolar-polar assemblages in the mid latitudes between 15 and 17 ka (Fig. 1b) documents a rapid response of the local fauna to the transient cooling and the subsequent warming caused by the Heinrich Event. It is possible that the direct response of planktonic foraminifera during the transition from the ice age (including the mid-latitude short-term cooling event) and the lagged and more complex response during the current warm period reflect faster response times of cold, species-poor assemblages compared with more species-rich warm-water assemblages. However, the most parsimonious explanation for the direct response would be that any lags in the assemblage response to climate change are shorter than the century-scale resolution of our time series and that the assemblage change during the current warm period does not reflect extinction debt^52^.

Thus, the question arises as to what the cause of the continued assemblage change could be. In this study, we use global mean surface temperature as a measure of climate change, but the assemblages responded to local rather than global mean forcing, as can be seen by the response to the mid-latitude short-term cooling event. In addition, global mean temperature is likely to be much less well correlated with local temperature during periods when temperature changes are small. This might partly explain the discrepancy between global mean temperature and the overall planktonic foraminifera response in the current warm period, but it cannot explain the progressive emergence of novel assemblages. However, temperature might not be the only driver of plankton biogeography especially at lower latitudes^8^, and food availability has also been shown to be important for temporal dynamics of planktonic foraminifera species^55,56^. In addition, other environmental factors such as the amplitude of seasonal temperature change or the degree of stratification of the water column, which changed during the current warm period^57^, might have contributed to the observed diversity patterns through the formation of new environmental vertical or seasonal niches.

Although it is difficult to decipher the exact cause of the continued change in the planktonic foraminifera assemblages during the current warm, stable period, one explanation could be a shift in the causes of species sorting in the planktonic foraminifera assemblages from abiotic-dominated causes (that is, temperature forcing) during the last deglaciation to more biotic-dominated causes (for example, changes in other plankton groups, food availability) during the current warm period. New direct and indirect ecological interactions between species of the same or other plankton groups might cause shifts in assemblage composition. Here we consider competition a less probable cause as no detectable evidence for interactions (that is, interspecific competition) within the planktonic foraminifera group itself has been found^58^. Instead, the continued change in planktonic foraminifera assemblages could have occurred due to a reorganization of their trophic interactions, reflecting changes in other aspects of the plankton community (for example, changes in the timing and composition of seasonal blooms, changes in predation pressure or exposure to new pathogens).

Notwithstanding the exact cause, the community dynamics during the current warm period were essential for the development of the present-day biogeography of planktonic foraminifera, including the distinct LDG with a tropical diversity dip^30^. We show that the flattening and ultimately the dip in tropical diversity in planktonic foraminifera evolved since the beginning of the current warm period at about 11 ka, at the end of the rapid deglacial warming (Fig. 5). We also show that the present-day shape of the LDG (Fig. 5a,c) is the result of species gains in the mid latitudes (Fig. 5b) combined with decreasing Shannon diversity in the tropics (Fig. 5d). The decreasing Shannon diversity indicates that few species became more dominant leading to more uneven assemblages and suggests that the equatorial region became progressively less hospitable to some species that inhabited the tropics during the LGM. It is therefore indeed possible that further warming will lead to species losses in this region, resulting in a tropical diversity crisis as predicted by macroecological modelling^30^. We also show that assemblage transformations occurred across the entire latitudinal gradient. Thus, the exact future shape of the LDG remains unclear because the continued warming could also lead to a loss of the surplus of species in the mid latitudes resulting from the asymmetry of gains versus losses^52^.

The establishment of novel planktonic foraminifera assemblages during the current warm period (Fig. 4) was the result of the poleward migration of species (Fig. 1b) in combination with the asymmetry of local immigration and extinction (Fig. 3). These asymmetrical shifts in species ranges induced by warming have also been observed and modelled in other marine taxa^7,10,12^. However, we show that the postglacial surplus of species in the mid latitudes (Fig. 3) was not lost by delayed local extinctions in these regions (extinction debt payment^52^) and that these novel assemblages are not a transient phenomenon of species response to global warming. Instead, we show that the compositional uniqueness of these assemblages persisted for millennia after the rapid deglacial warming. This provides observational constraints for modelling, indicating that the projected future warming could also lead to the assembly of long-lasting novel marine communities^10,12^ with potentially important consequences for key ecosystem functions.

## Methods

### Data

The community change analyses were based on 25 planktonic foraminifera assemblage time series covering the past 24 kyr, with an average resolution of 0.60 kyr, ranging from 0.04 to 1.31 kyr (Extended Data Table 1). Throughout this contribution, age information is provided in calibrated radiocarbon years, so 0 ka is 1950 Common Era. The series were selected from among 198 records situated in the North Atlantic Ocean and adjacent seas initially identified in public databases as containing planktonic foraminifera assemblage counts spanning the transition from the last ice age to the current warm period. Of these, only time series where the entire assemblages had been counted were used and further limited to time series that recorded the entire time period of interest, that is, beginning at least at 23 ka and ranging to at least 3 ka with a resolution below 1.5 kyr to resolve millennial-scale climate events. The remaining 25 time series cover the full latitudinal and thermal gradient in the North Atlantic Ocean (Fig. 1a). For the 9 sites included in the PALMOD 130k marine palaeoclimate data synthesis V1.1^19^, we used their provided revised age models based on radiocarbon ages and benthic foraminifera oxygen isotope data which were manually tuned to regional benthic foraminifera oxygen isotope stacks^59^. Their radiocarbon ages were re-calibrated with the IntCal13 calibration curve^60^ using reservoir ages based on a comprehensive ocean general circulation model^61^. For the 16 sites not included in the PALMOD 130k marine palaeoclimate data synthesis V1.1^19^, the same approach as in ref. ^19^ was used to revise the published age models to ensure the comparability of all analysed sites (Extended Data Table 1). The age model revisions were conducted with PaleoDataView^62^.

**Extended Data Fig. 2:**
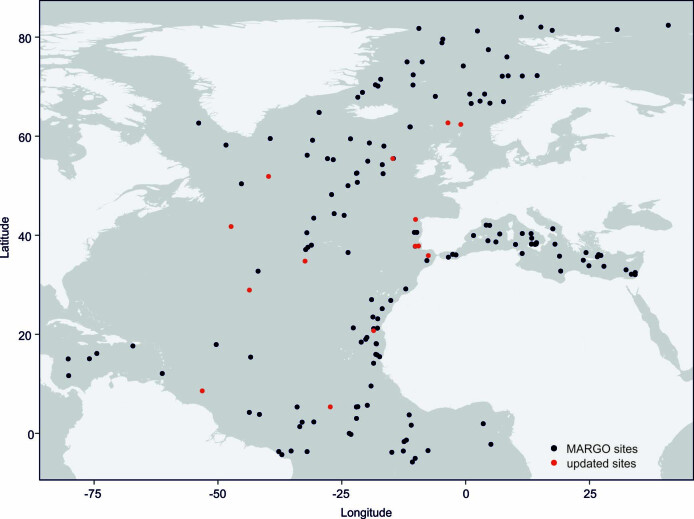
Overview map of LGM dataset. Updated LGM compilation (1083 samples from 173 sites) used to assess the LGM assemblage analogues. This compilation is based on a MARGO subset^36^ that was updated with samples from the time series used in this study that belonged to the LGM interval (194 samples from 14 sites). The geographical extent was restricted to the North Atlantic Ocean (including the Mediterranean Sea and the Arctic Ocean) and the same latitudinal extent as the 25 time series used in this study (that is, up to a latitude of 6°S).

Assemblage composition of planktonic foraminifera in the LGM ocean was analysed using a regional North Atlantic subset of the MARGO compilation^36^, covering the same latitudinal range as the 25 time series used in this study (that is, 90° N to 6° S). Samples from the time series of this study that belonged to the LGM interval but were not present in the MARGO synthesis (that is, published after 2005) were also added to the LGM dataset (194 samples from 14 sites). We used the LGM time interval as defined in ref. ^63^ and in the MARGO compilation^36^ of 19–23 ka. In total, the updated LGM compilation consists of 1,083 assemblage compositions from 173 unique sites (Extended Data Fig. 2). The global mean surface temperature (Fig. 2c) used for the comparison with the overall response of the planktonic foraminifera assemblages is the result of a data assimilation approach that combines 539 proxy records with independent model information^64^. The temperature anomalies were referenced to the mean of the past two millennia (0–2 ka).

All planktonic foraminifera assemblage count data used here were harmonized taxonomically following ref. ^38^. Species not reported in the time series data were assumed to be absent (that is, zero abundance). We merged *Globigerinoides ruber ruber* and *Globigerinoides ruber albus* because some studies only reported them together as *Globigerinoides ruber*. Also, P/D intergrades (an informal category of morphological intermediates between *Neogloboquadrina incompta* and *Neogloboquadrina dutertrei*) were merged with *Neogloboquadrina incompta*. In total, 41 species of planktonic foraminifera were included in our study (Extended Data Table 2).

### Spatio-temporal compositional dissimilarity

To visualize which time periods and regions in the oceans have similar species composition (Fig. 1b), we calculated the compositional dissimilarity between all pairwise combinations of all samples in the 25 time series (1,840 samples in total). The compositional dissimilarity was calculated using the Morisita-Horn (M-H) index^65^: $C = 1 - \frac{{2 \times \mathop {\sum }\nolimits_{i = 1}^S \left( {x_i \times y_i} \right)}}{{\mathop {\sum }\nolimits_{i = 1}^S x_i^2 + \mathop {\sum }\nolimits_{i = 1}^S y_i^2}}$, where *S* is the total number of species in both samples, and *x*_*i*_ and *y*_*i*_ are the relative abundances of the *i*-th species in both samples. The M-H index is a turnover measure based on distance that is relatively independent of sample size and robust to under-sampling^66^. The measure ranges from 0 to 1, with 0 indicating an identical composition of the two samples and 1 indicating no shared species. We then applied a principal component analysis (PCA) to the compositional dissimilarity matrix to reduce its dimensionality and visualize the spatio-temporal evolution of assemblage composition. The first three PC axes explained more than 97% of the variance and we assigned an RGB value to each of these axes (PC1 blue, PC2 red, PC3 green^67^). As a result, each sample of our study had an RGB value related to its projection in the PC dissimilarity space. These RGB values were then plotted in a Hovmoller-like plot (Fig. 1b) where similar colours in the grid correspond to similar species compositions.

### PCA on species composition

To determine the temporal pattern of compositional change in the analysed planktonic foraminifera time series, we applied a PCA for each time series on the species assemblage data and extracted for each time series the axis that explains most of the variance in the assemblage data (PC1). We fitted linear models through all PC1 axes to check and, if necessary, change the polarization to align all PC1 axes in the same direction. To adjust for different resolutions of the individual records, we interpolated the PC1 scores at 0.5 kyr bins and restricted the interpolated data to the interval that is covered by all time series (2.5–23 ka) to prevent edge effects. Because the shape of the faunal trends at all sites was similar, we visualized the overall trend of faunal response among the 25 time series by a polynomial regression using a locally estimated scatterplot smoothing (LOESS, using standard settings) on the interpolated individual PC1 axes scores (Fig. 2a).

### Species gains and losses

**Extended Data Fig. 3:**
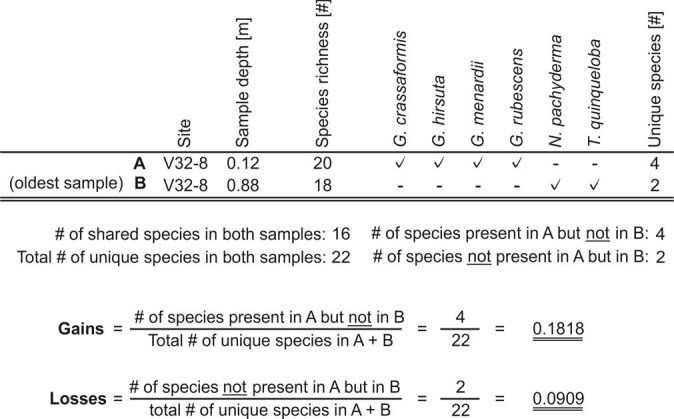
Example for gains and losses calculation. This example shows the calculation of the gains and losses values for Sample A (site V32-8; 0.12 m depth). For the gains and losses calculation, all samples in a time series are always compared to the oldest sample in that time series (here Sample B; site V32-8; 0.88 m depth). Sample A contains 20 species with 4 (*G. crassaformis*, *G. hirsuta*, *G. menardii* and *G. rubescens*) not being present in Sample B, whereas Sample B (18 species) contains 2 species (*N. pachyderma* and *T. quinqueloba*) not present in sample A. Both samples share 16 species. Gains are calculated as the proportion of the number of species present in Sample A but not in Sample B relative to the total number of species in both samples pooled together (22 species: 16 shared plus 6 unique species) resulting in a gain value of 0.1818. Losses are calculated as the number of species not present in Sample A but present in Sample B relative to the total number of species in both samples resulting in a loss value of 0.0909. Sample A and B are also highlighted in Extended Data Fig. 4c.

**Extended Data Fig. 4:**
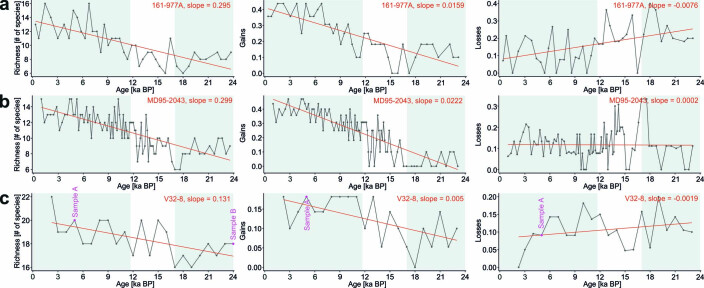
Example of fitted linear models. To quantify the rates of biodiversity change we calculated the slope of fitted linear models for species richness, gains and losses. Here, the fitted linear models are shown for three exemplary sites: 161-977A (**a**), MD95-2043 (**b**) and V32-8 (**c**). Species richness is the absolute number of species in each sample. A positive slope in richness indicates an increase in the number of species since the LGM and a negative slope means a decrease. Gains and losses are given as the proportion between the gained/lost species in each sample compared to the oldest sample in each time series relative to the total number of species in both samples pooled together (see Extended Data Fig. 3); since gains and losses are given as proportions, they are unitless. For gains (losses), a positive slope indicates that the number of species gained (lost) at a given site increased over time. Time series in which the number of gained (lost) species is decreasing through time show a negative slope. In other words, a positive slope of species gains (losses) means that the richness is increasing (decreasing) continuously through time as species gains (losses) are accumulating through time. A slope of species gains/losses equals zero means that the richness remains constant over time as no species gains/losses are accumulated through time. Red lines correspond to the fitted linear models and the slopes are given in the upper right corner of each panel. **c**, also shows the temporal location of Sample A and B used in Extended Data Fig. 3.

To analyse local biodiversity change, we first calculated species richness (Fig. 3a,b) at every location and time step and the proportion of species gained (Fig. 3c,d) and lost (Fig. 3e,f) compared to the LGM (oldest sample in the time series). Species gains and losses were calculated for each sample in a time series as the proportion of species either gained or lost in comparison to the oldest sample in that time series relative to the total number of species observed in both samples pooled together, taking species identity into account^10^ (see Extended Data Fig. 3). We then calculated the slopes of fitted linear models for species richness, gains and losses to quantify the rates of biodiversity change (see Extended Data Fig. 4). The rate of richness change is given in species per unit time and the rates of gains and losses change are given in the proportion of gained or lost species (compared to the oldest sample in the time series) per unit time over the entire length of the time series. A positive slope in richness indicates an increase in the number of species through time and a negative slope means a decrease. For gains (losses), a positive slope indicates that the proportion of species gained (lost) at a given site compared to the oldest sample in the time series increases through time, meaning that species gains (losses) are accumulated through time leading to an increase (decrease) in species richness. Time series where the proportion of gained (lost) species is decreasing through time show a negative slope.

### No-analogues assemblages

**Extended Data Fig. 5:**
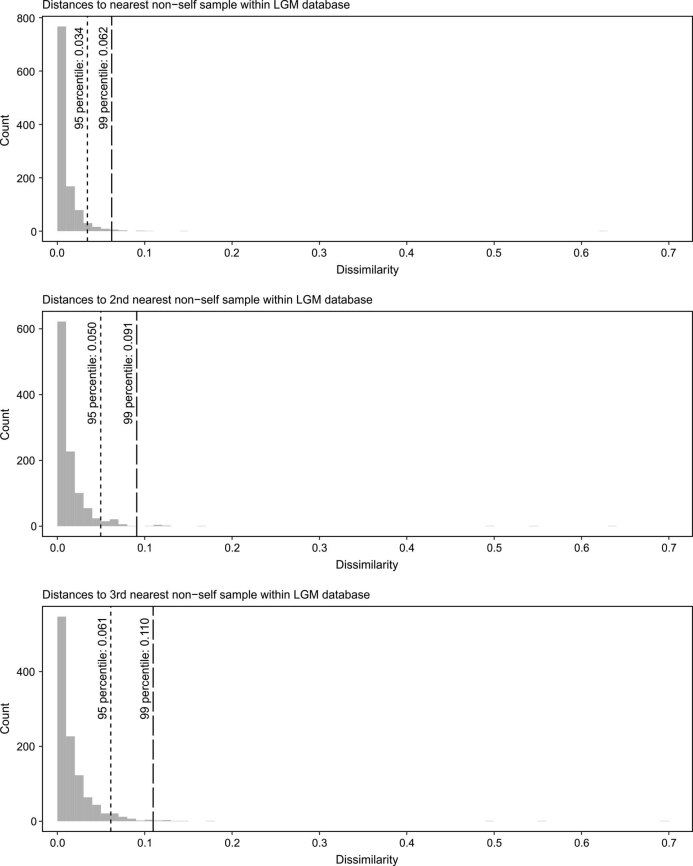
Histogram of nearest non-self analogues. Distance to nearest (as well as 2nd and 3rd nearest) non-self analogue within LGM compilation (see Extended Data Fig. 2). 99 % of the LGM samples have a nearest analogue with a dissimilarity of less than 0.06.

To investigate the potential formation of new assemblages during the planktonic foraminifera response to deglacial warming after the LGM, we calculated for each assemblage in the time series the compositional dissimilarity (M-H index) to all the assemblages from the LGM compilation (see Data section above). We then obtained the distance to the nearest analogue from the minimum dissimilarity. Figure 4 shows these minima gridded in a Hovmoller-like plot. To judge whether the observed minimum M-H distance indicated a no-analogue assemblage, we calculated M-H index values for each of the LGM compilation samples relative to the remaining samples in the compilation, thus obtaining threshold values for M-H index dissimilarities that do not necessarily represent no-analogue faunas and could occur by chance. We calculated the 95 and 99 percentiles of the M-H distances to the nearest (as well as 2nd- and 3rd-nearest) non-self analogue within the LGM compilation (Extended Data Fig. 2) and compared it with the observed no-analogue values. We found that 99% of the LGM samples have a nearest analogue with a dissimilarity of less than 0.06 (as well as 2nd-nearest analogue of <0.09 and 3rd-nearest analogue of <0.11) within the LGM dataset (Extended Data Fig. 5). Therefore, we claim that the dissimilarities of 0.15–0.25 that we observed in the mid latitudes in the Holocene samples (Fig. 4) are significantly higher than could be expected to happen by chance, pointing to changing assemblages with no LGM analogues.

### LDG through time

To visualize the temporal evolution of the planktonic foraminifera LDG in the North Atlantic Ocean, we pooled all samples from each time series within millennial bins and calculated the number of species (richness) and the Shannon entropy^68^, an abundance-based diversity metric: $H_S = - \mathop {\sum}\nolimits_{i = 1}^S {p_i \times \log p_i}$, where *S* is the number of species at a specific site and *p*_*i*_ is the relative abundance of the *i*-th species. Because relative abundances are always between 0 and 1, the higher the metric, the more diverse the assemblage. The latitudinal gradients of species richness and Shannon diversity were then visualized for each millennium by polynomial regressions using LOESS (Fig. 5a,c).

To understand when and where diversity change occurred during the past 24 kyr, we calculated for each sample, the difference between its richness and Shannon diversity and the mean LGM richness and Shannon diversity of the site. The mean LGM richness and Shannon diversity were calculated across all samples in a given time series that fall within 19–23 ka. These differences were then gridded in Hovmoller-like plots with a grid cell resolution in time and space of 1 kyr and 2.5° (Fig. 5b,d).

### R packages

All statistical analyses were performed with R version 4.1.3^69^ using the tidyverse^70^ and the janitor^71^ packages for cleaning and importing the data; vegan^72^ and codyn^73^ for beta diversity and community structure analyses; rioja^74^ for the nearest-analogue analysis; FactoMineR^75^ for the PCA analysis; and ggplot2^76^, raster^77^ and vidiris^78^ for the plots.

### Reporting summary

Further information on research design is available in the Nature Research Reporting Summary linked to this article.

### Supplementary information

Supplementary information Supplementary Reference List.

Reporting Summary

## Figures and Tables

**Extended Data Table 1:**
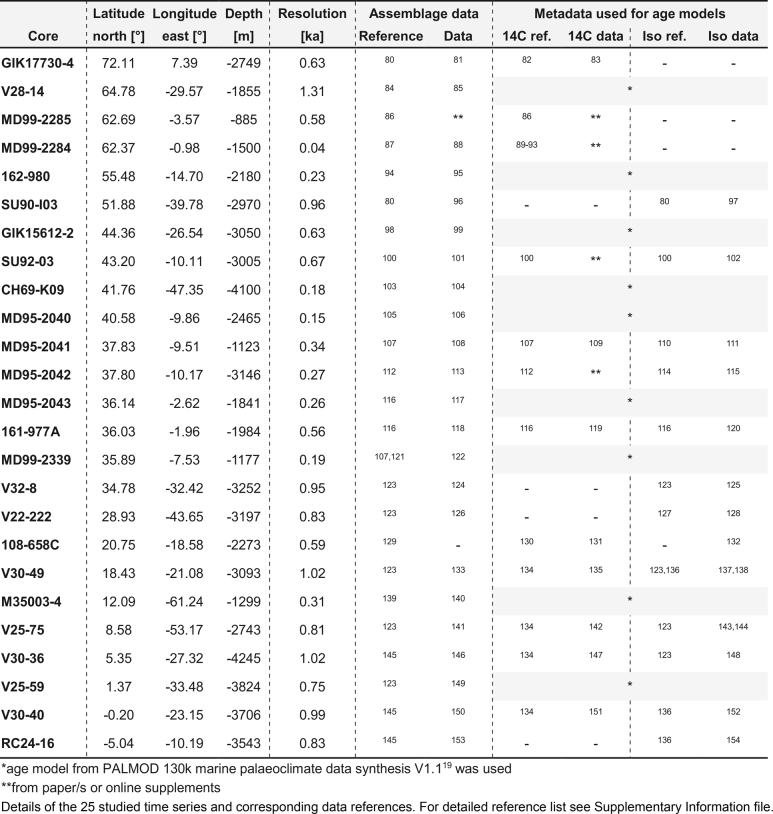
Overview of time series

**Extended Data Table 2:**
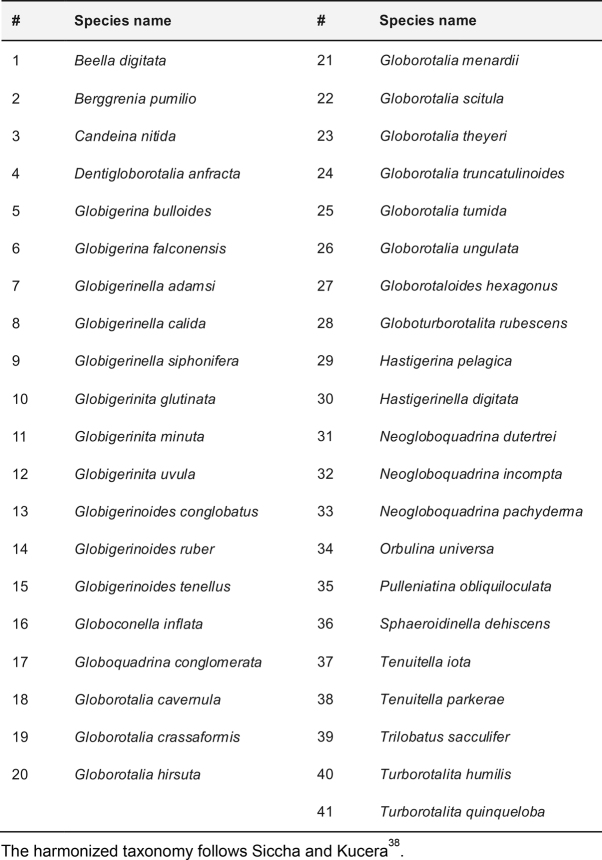
Species list of planktonic foraminifera

## Data Availability

All data used and analysed during the current study are publicly available in the PANGAEA and NOAA National Centers for Environmental Information repositories. For information on links and paper references to individual assemblage datasets, see Extended Data Table 1. MARGO data that are used for the regional North Atlantic LGM dataset are available on PANGAEA (Atlantic Ocean: 10.1594/PANGAEA.227329, Mediterranean: 10.1594/PANGAEA.227306 and Pacific: 10.1594/PANGAEA.227327). Modern global mean surface temperature and globally resolved surface temperature since the LGM are available at NOAA (https://www.ncei.noaa.gov/access/world-ocean-atlas-2018/bin/woa18.pl and https://www.ncei.noaa.gov/pub/data/paleo/reconstructions/osman2021/). Taxonomically harmonized assemblage data are available at 10.5281/zenodo.6948750.

## Code availability

The R code used to generate the results of this study is available at 10.5281/zenodo.6948750.
