# Supplementary material for: Plankton response to global warming is characterized by non-uniform shifts in assemblage composition since the last ice age
Source: Nat Ecol Evol. 2022 Oct 10;6(12):1871–80. doi: 10.1038/s41559-022-01888-8 (PMC9715429; doi:10.1038/s41559-022-01888-8)
Supplement: Supplementary file 1 — Supplementary Reference List. [file 41559_2022_1888_MOESM1_ESM.pdf]

nature ecology & evolution

Article

https://doi.org/10.1038/s41559-022-01888-8

Plankton response to global warming is characterized by non-uniform shifts in assemblage composition since the last ice age

In the format provided by the authors and unedited

SUPPLEMENTARY INFORMATION

Strack, T., Jonkers, L., Rillo, M.C., Hillebrand, H., and Kucera, M., *submitted 2022*, Plankton response to global warming is characterized by non-uniform shifts in assemblage composition since the last ice age.

1. Supplementary Reference List

References given here correspond to the references given in Extended Data Table 1. Included are references and data links for the planktonic foraminifera assemblage data used in this study as well as references and metadata links for 14C and isotope data that have been used for the age models.

80 Schulz, H. *Meeresoberflächentemperaturen vor 10.000 Jahren - Auswirkungen des frühholozänen Insolationsmaximums.* Doctoral dissertation thesis, Christian-Albrechts-Universität Kiel, (1995).

81 Schulz, H. Planktic foraminifera assemblage in sediment core GIK17730-4. *PANGAEA*, doi:https://doi.org/10.1594/PANGAEA.134132 (1995).

82 Sarnthein, M. *et al.* Variations in Atlantic surface ocean paleoceanography, 50°-80°N: A time-slice record of the last 30,000 years. *Paleoceanography* **10**, 1063-1094, doi:10.1029/95pa01453 (1995).

83 Sarnthein, M. Age determination of sediment core GIK17730-4. *PANGAEA*, doi:https://doi.org/10.1594/PANGAEA.134194 (2003).

84 CLIMAP Project Members. Seasonal reconstruction of the earth's surface at the last glacial maximum. *Geological Society of America, Map and Chart Series* **36**, 18pp (1981).

85 CLIMAP Project Members. Planktic foraminifera counts of sediment core V28-14. *PANGAEA*, doi:https://doi.org/10.1594/PANGAEA.727379 (2009).

86 Wary, M. *et al.* The southern Norwegian Sea during the last 45 ka: hydrographical reorganizations under changing ice-sheet dynamics. *J. Quat. Sci.* **32**, 908-922, doi:10.1002/jqs.2965 (2017).

87 Eldevik, T. *et al.* A brief history of climate – the northern seas from the Last Glacial Maximum to global warming. *Quat. Sci. Rev.* **106**, 225-246, doi:10.1016/j.quascirev.2014.06.028 (2014).

88 Dokken, T., Andersson, C. & Risebrobakken, B. Relative abundance of planktic foraminifera and calculated SSTs and SST anomaly (0-25.5 ka BP) in sediment core MD99-2284. *PANGAEA*, doi:https://doi.org/10.1594/PANGAEA.846924 (2015).

89 Bakke, J. *et al.* Rapid oceanic and atmospheric changes during the Younger Dryas cold period. *Nature Geoscience* **2**, 202-205, doi:10.1038/ngeo439 (2009).

90 Dokken, T. M., Nisancioglu, K. H., Li, C., Battisti, D. S. & Kissel, C. Dansgaard-Oeschger cycles: Interactions between ocean and sea ice intrinsic to the Nordic seas. *Paleoceanography* **28**, 491-502, doi:10.1002/palo.20042 (2013).

91 Rasmussen, S. O. *et al.* A new Greenland ice core chronology for the last glacial termination. *J. Geophys. Res.* **111**, doi:10.1029/2005jd006079 (2006).

92 Risebrobakken, B. *et al.* Early Holocene temperature variability in the Nordic Seas: The role of oceanic heat advection versus changes in orbital forcing. *Paleoceanography* **26**, doi:10.1029/2011pa002117 (2011).

93 Svensson, A. *et al.* A 60 000 year Greenland stratigraphic ice core chronology. *Clim. Past* **4**, 47-57, doi:10.5194/cp-4-47-2008 (2008).

94 Benway, H. M., McManus, J. F., Oppo, D. W. & Cullen, J. L. Hydrographic changes in the eastern subpolar North Atlantic during the last deglaciation. *Quat. Sci. Rev.* **29**, 3336-3345, doi:10.1016/j.quascirev.2010.08.013 (2010).

95 Benway, H. M., McManus, J. F., Oppo, D. W. & Cullen, J. L. *Eastern Subpolar North Atlantic Stable Isotope, Mg/Ca and SST Data During the Last Deglaciation*, <https://www1.ncdc.noaa.gov/pub/data/paleo/contributions_by_author/benway2010/benway2010-fauna.txt> (2013).

96 Schulz, H. Planktic foraminifera assemblage in sediment core SU90-I03. *PANGAEA*, doi:https://doi.org/10.1594/PANGAEA.134150 (1995).

97 Schulz, H. Stable isotope analysis on planktic foraminifera in sediment core SU90-I03. *PANGAEA*, doi:https://doi.org/10.1594/PANGAEA.107751 (1995).

98 Kiefer, T. *Produktivität und Temperaturen im subtropischen Nordatlantik: zyklische und abrupte Veränderunge im späten Quartär* Doctoral dissertation thesis, Christian-Albrechts-Universität Kiel, (1998).
99 Kiefer, T. & Sarnthein, M. Distribution of planktic foraminifers of sediment core GIK15612-2. *PANGAEA*, doi:https://doi.org/10.1594/PANGAEA.202139 (1998).
100 Salgueiro, E. *et al.* Temperature and productivity changes off the western Iberian margin during the last 150 ky. *Quat. Sci. Rev.* **29**, 680-695, doi:10.1016/j.quascirev.2009.11.013 (2010).
101 Salgueiro, E. *et al.* Planktonic foraminiferal abundances, and sea surface and export production reconstructions of sediment core SU92-03. *PANGAEA*, doi:https://doi.org/10.1594/PANGAEA.743086 (2010).
102 Salgueiro, E. *et al.* Stable oxygen isotope record of planktonic and benthic foraminifera of sediment core SU92-03. *PANGAEA*, doi:https://doi.org/10.1594/PANGAEA.743085 (2010).
103 Labeyrie, L. *et al.* Temporal variability of the surface and deep waters of the North West Atlantic Ocean at orbital and millenial scales. *Mechanisms of Global Climate Change at Millennial Time Scales*, 77-98, doi:10.1029/gm112p0077 (1999).
104 Labeyrie, L. D. *et al.* Planktonic foraminifera in sediment core CH69-K09. *PANGAEA*, doi:https://doi.org/10.1594/PANGAEA.881450 (2017).
105 de Abreu, L., Shackleton, N. J., Schönfeld, J., Hall, M. & Chapman, M. Millennial-scale oceanic climate variability off the Western Iberian margin during the last two glacial periods. *Mar. Geol.* **196**, 1-20, doi:10.1016/s0025-3227(03)00046-x (2003).
106 de Abreu, L., Shackleton, N. J., Schönfeld, J., Hall, M. A. & Chapman, M. R. Planktic foraminifera counts of sediment core MD95-2040. *PANGAEA*, doi:https://doi.org/10.1594/PANGAEA.66714 (2003).
107 Voelker, A. H. L. & de Abreu, L. in *Abrupt Climate Change: Mechanisms, Patterns, and Impacts Geophysical Monograph Series* (eds H Rashid, L Polyak, & E Mosley-Thompson) 15-37 (AGU, 2011).
108 Voelker, A. H. L. & de Abreu, L. Distribution of planktonic foraminifera in sediment core MD95-2041. *PANGAEA*, doi:https://doi.org/10.1594/PANGAEA.737130 (2011).
109 Voelker, A. H. L. & de Abreu, L. Stable oxygen and carbon isotope ratios of Globigerina bulloides of sediment core MD95-2041. *PANGAEA*, doi:https://doi.org/10.1594/PANGAEA.737129 (2011).
110 Voelker, A. H. L., de Abreu, L., Schönfeld, J., Erlenkeuser, H. & Abrantes, F. Hydrographic conditions along the western Iberian margin during marine isotope stage 2. *Geochemistry, Geophysics, Geosystems* **10**, Q12U08, doi:10.1029/2009gc002605 (2009).
111 Voelker, A. H. L., de Abreu, L., Schönfeld, J., Erlenkeuser, H. & Abrantes, F. F. (Table 6) Age model for sediment core MD95-2041. *PANGAEA*, doi:https://doi.org/10.1594/PANGAEA.733062 (2009).
112 Chabaud, L., Sánchez Goñi, M. F., Desprat, S. & Rossignol, L. Land–sea climatic variability in the eastern North Atlantic subtropical region over the last 14,200 years: Atmospheric and oceanic processes at different timescales. *Holocene* **24**, 787-797, doi:10.1177/0959683614530439 (2014).
113 Rossignol, L. Planktonic foraminifera counts of sediment core MD95-2042 off the Iberian margin for the past 60 ka. *PANGAEA*, doi: https://doi.org/10.1594/PANGAEA.946601 (2022).
114 Cayre, O., Lancelot, Y., Vincent, E. & Hall, M. A. Paleoceanographic reconstructions from planktonic foraminifera off the Iberian Margin: Temperature, salinity, and Heinrich events. *Paleoceanography* **14**, 384-396, doi:10.1029/1998pa900027 (1999).
115 Cayre, O., Vincent, E., Lancelot, Y. & Hall, M. A. Stable oxygen and carbon isotope ratios of Globigerina bulloides in sediment core MD95-2042 of the Iberian Margin, North Atlantic. *PANGAEA*, doi:https://doi.org/10.1594/PANGAEA.60829 (1999).
116 Pérez-Folgado, M. *et al.* Western Mediterranean planktonic foraminifera events and millennial climatic variability during the last 70 kyr. *Mar. Micropaleontol.* **48**, 49-70, doi:10.1016/S0377-8398(02)00160-3 (2003).
117 Pérez-Folgado, M. *et al.* (Appendix 2) Distribution of planktonic foraminifera in the last 70 kyr of sediment core MD95-2043 from the western Mediterranean Sea. *PANGAEA*, doi:https://doi.org/10.1594/PANGAEA.678286 (2003).
118 Pérez-Folgado, M. *et al.* (Appendix 1) Distribution of planktonic foraminifera in sediment of the last 70 kyr of ODP Core 161-977A from the western Mediterranean Sea. *PANGAEA*, doi:https://doi.org/10.1594/PANGAEA.678285 (2003).
119 Pérez-Folgado, M. *et al.* (Table 1) Age model of ODP Core 161-977A from the western Mediterranean Sea. *PANGAEA*, doi:https://doi.org/10.1594/PANGAEA.678288 (2003).

120 Pérez-Folgado, M. *et al.* (Appendix 3) Stable oxygen isotope ratios of Globigerina bulloides during the last 70 kyr of ODP Core 161-977A from the western Mediterranean Sea. *PANGAEA*, doi:https://doi.org/10.1594/PANGAEA.678287 (2003).
121 Eynaud, F. *et al.* Position of the Polar Front along the western Iberian margin during key cold episodes of the last 45 ka. *Geochem. Geophys. Geosyst.* **10**, Q07U05, doi:10.1029/2009gc002398 (2009).
122 Voelker, A. H. L., Schönfeld, J., Lebreiro, S. M. & Abrantes, F. F. Relative abundances of planktonic foraminifera in sediment core MD99-2339. *PANGAEA*, doi:https://doi.org/10.1594/PANGAEA.733102 (2010).
123 Mix, A. C. *Late Quaternary paleoceanography of the Atlantic Ocean: Foraminiferal faunal and stable-isotopic evidence.* Doctoral dissertation thesis, Columbia University, (1986).
124 Mix, A. C. Percentages of planktonic foraminifera species in sediment core V32-8. *PANGAEA*, doi:https://doi.org/10.1594/PANGAEA.355352 (2006).
125 Mix, A. C. Stable oxygen isotope ratios of G. ruber from sediment core V32-8. *PANGAEA*, doi:https://doi.org/10.1594/PANGAEA.55808 (1999).
126 Mix, A. C. Percentages of planktonic foraminifera species in sediment core V22-222. *PANGAEA*, doi:https://doi.org/10.1594/PANGAEA.355340 (2006).
127 Mix, A. C., Ruddiman, W. F. & McIntyre, A. Late Quaternary paleoceanography of the Tropical Atlantic, 1: Spatial variability of annual mean sea-surface temperatures, 0-20,000 years B.P. *Paleoceanography* **1**, 43-66, doi:10.1029/pa001i001p00043 (1986).
128 Mix, A. C., Ruddiman, W. F. & McIntyre, A. (Table 3) Stable oxygen isotope ratios of Globigerinoides ruber from sediment core V22-222. *PANGAEA*, doi:https://doi.org/10.1594/PANGAEA.52244 (1986).
129 Haslett, S. K. & Smart, C. W. Late Quaternary upwelling off tropical NW Africa: new micropalaeontological evidence from ODP Hole 658C. *J. Quat. Sci.* **21**, 259-269, doi:10.1002/jqs.970 (2006).
130 deMenocal, P. *et al.* Abrupt onset and termination of the African Humid Period: rapid climate responses to gradual insolation forcing. *Quat. Sci. Rev.* **19**, 347-361, doi:10.1016/S0277-3791(99)00081-5 (2000).
131 deMenocal, P. B. *et al.* Age determination of ODP Hole 108-658C (Table 1). *PANGAEA*, doi:https://doi.org/10.1594/PANGAEA.742958 (2000).
132 Knaack, J.-J. & Sarnthein, M. Stable isotopes of foraminifera of ODP Hole 108-658C. *PANGAEA*, doi:https://doi.org/10.1594/PANGAEA.227736 (2005).
133 Mix, A. C. Percentages of planktonic foraminifera species in sediment core V30-49. *PANGAEA*, doi:https://doi.org/10.1594/PANGAEA.355350 (2006).
134 Mix, A. C. & Ruddiman, W. F. Structure and timing of the last deglaciation: Oxygen-isotope evidence *Quat. Sci. Rev.* **4**, 59-108, doi:10.1016/0277-3791(85)90015-0 (1985).
135 CLIMAP Project Members. Radiocarbon age determinations on sediment core V30-49. *PANGAEA*, doi:https://doi.org/10.1594/PANGAEA.186299 (2004).
136 McIntyre, A., Ruddiman, W. F., Karlin, K. & Mix, A. C. Surface water response of the Equatorial Atlantic Ocean to orbital forcing *Paleoceanography* **4**, 19-55, doi:10.1029/PA004i001p00019 (1989).
137 Mix, A. C. Stable isotope ratios of G. sacculifer from sediment core V30-49. *PANGAEA*, doi:https://doi.org/10.1594/PANGAEA.112954 (2003).
138 Ruddiman, W. F. Stable isotopes of sediment core V30-49 (specmap.012). *PANGAEA*, doi:https://doi.org/10.1594/PANGAEA.52125 (1997).
139 Hüls, C. M. *Millennial-scale SST variability as inferred from planktonic foraminifera census counts in the western subtropical Atlantic.* Doctoral dissertation thesis, GEOMAR Report 95, GEOMAR Research Center for Marine Geoscience, Christian Albrechts University in Kiel, (2000).
140 Hüls, C. M. Distribution of planktic foraminifera of sediment core M35003-4. *PANGAEA*, doi:https://doi.org/10.1594/PANGAEA.55756 (1999).
141 Mix, A. C. Percentages of planktonic foraminifera species in sediment core V25-75. *PANGAEA*, doi:https://doi.org/10.1594/PANGAEA.355345 (2006).
142 CLIMAP Project Members. Radiocarbon age determinations on sediment core V25-75. *PANGAEA*, doi:https://doi.org/10.1594/PANGAEA.186282 (2004).
143 Mix, A. C. Stable isotope ratios of G. sacculifer from sediment core V25-75. *PANGAEA*, doi:https://doi.org/10.1594/PANGAEA.112950 (2003).
144 Mix, A. C. Stable oxygen isotope ratios of U. peregrina from sediment core V25-75. *PANGAEA*, doi:https://doi.org/10.1594/PANGAEA.55807 (1999).

145 Mix, A. C., Morey, A. E., Pisias, N. G. & Hostetler, S. W. Foraminiferal faunal estimates of paleotemperature: Circumventing the No-analog problem yields cool Ice Age tropics. *Paleoceanography* **14**, 350-359, doi:10.1029/1999pa900012 (1999).
146 Mix, A. C., Morey, A. E., Pisias, N. G. & Hostetler, S. W. Distribution of planktic foraminifers of core V30-36. *PANGAEA*, doi:https://doi.org/10.1594/PANGAEA.61069 (1999).
147 CLIMAP Project Members. Radiocarbon age determinations on sediment core V30-36. *PANGAEA*, doi:https://doi.org/10.1594/PANGAEA.186296 (2004).
148 Mix, A. C. Stable isotope ratios of G. sacculifer from sediment core V30-36. *PANGAEA*, doi:https://doi.org/10.1594/PANGAEA.112952 (2003).
149 Mix, A. C. Percentages of planktonic foraminifera species in sediment core V25-59. *PANGAEA*, doi:https://doi.org/10.1594/PANGAEA.355343 (2006).
150 Mix, A. C., Morey, A. E., Pisias, N. G. & Hostetler, S. W. Distribution of planktic foraminifers of core V30-40. *PANGAEA*, doi:https://doi.org/10.1594/PANGAEA.61070 (1999).
151 CLIMAP Project Members. Radiocarbon age determinations on sediment core V30-40. *PANGAEA*, doi:https://doi.org/10.1594/PANGAEA.186297 (2004).
152 McIntyre, A. & Imbrie, J. D. Stable isotopes of sediment core V30-40 (specmap.010). *PANGAEA*, doi:https://doi.org/10.1594/PANGAEA.56361 (2000).
153 Mix, A. C., Morey, A. E., Pisias, N. G. & Hostetler, S. W. Distribution of planktic foraminifers of core RC24-16. *PANGAEA*, doi:https://doi.org/10.1594/PANGAEA.61066 (1999).
154 Imbrie, J. D. & McIntyre, A. Stable isotope analysis on sediment core RC24-16 (specmap.004). *PANGAEA*, doi:https://doi.org/10.1594/PANGAEA.56373 (2000).
