# Supplementary material for: Plankton response to global warming is characterized by non-uniform shifts in assemblage composition since the last ice age
Source: Nat Ecol Evol. 2022 Oct 10;6(12):1871–80. doi: 10.1038/s41559-022-01888-8 (PMC9715429; doi:10.1038/s41559-022-01888-8)
Supplement: Supplementary file 2 — Reporting Summary [file 41559_2022_1888_MOESM2_ESM.pdf]

nature portfolio

Corresponding author(s): Tonke Strack

Last updated by author(s): Aug 1, 2022

Reporting Summary

Nature Portfolio wishes to improve the reproducibility of the work that we publish. This form provides structure for consistency and transparency in reporting. For further information on Nature Portfolio policies, see our Editorial Policies and the Editorial Policy Checklist.

Statistics

For all statistical analyses, confirm that the following items are present in the figure legend, table legend, main text, or Methods section.

n/a	Confirmed	
☐	☒	The exact sample size (*n*) for each experimental group/condition, given as a discrete number and unit of measurement
☒	☐	A statement on whether measurements were taken from distinct samples or whether the same sample was measured repeatedly
☐	☒	The statistical test(s) used AND whether they are one- or two-sided<br>*Only common tests should be described solely by name; describe more complex techniques in the Methods section.*
☒	☐	A description of all covariates tested
☒	☐	A description of any assumptions or corrections, such as tests of normality and adjustment for multiple comparisons
☒	☐	A full description of the statistical parameters including central tendency (e.g. means) or other basic estimates (e.g. regression coefficient) AND variation (e.g. standard deviation) or associated estimates of uncertainty (e.g. confidence intervals)
☒	☐	For null hypothesis testing, the test statistic (e.g. *F*, *t*, *r*) with confidence intervals, effect sizes, degrees of freedom and *P* value noted<br>*Give P values as exact values whenever suitable.*
☒	☐	For Bayesian analysis, information on the choice of priors and Markov chain Monte Carlo settings
☒	☐	For hierarchical and complex designs, identification of the appropriate level for tests and full reporting of outcomes
☒	☐	Estimates of effect sizes (e.g. Cohen's *d*, Pearson's *r*), indicating how they were calculated

Our web collection on statistics for biologists contains articles on many of the points above.

Software and code

Policy information about availability of computer code

Data collection	no software used for collection
Data analysis	R version 4.1.3; publicly available packages listed in methods

For manuscripts utilizing custom algorithms or software that are central to the research but not yet described in published literature, software must be made available to editors and reviewers. We strongly encourage code deposition in a community repository (e.g. GitHub). See the Nature Portfolio guidelines for submitting code & software for further information.

Data

Policy information about availability of data

All manuscripts must include a data availability statement. This statement should provide the following information, where applicable:

- Accession codes, unique identifiers, or web links for publicly available datasets
- A description of any restrictions on data availability
- For clinical datasets or third party data, please ensure that the statement adheres to our policy

All data used and analysed during the current study are publicly available in the PANGAEA and NOAA National Centers for Environmental Information repositories (except assemblage abundance data for 108-658C). For information on links and paper references to individual assemblage datasets see Extended Data Table 1. Abundance data of 108-658C is an unpublished dataset used with the permission of the authors.
MARGO data that are used for the regional North Atlantic LGM data set are available on PANGAEA (Atlantic Ocean: https://doi.pangaea.de/10.1594/PANGAEA.227329, Mediterranean: https://doi.pangaea.de/10.1594/PANGAEA.227306 and Pacific: https://doi.pangaea.de/10.1594/PANGAEA.227327). Modern global mean surface temperature and globally resolved surface temperature since the LGM are available at NOAA (https://www.ncei.noaa.gov/access/world-ocean-atlas-2018/bin/woa18.pl and https://www.ncei.noaa.gov/pub/data/paleo/reconstructions/osman2021/).

Taxonomically harmonized assemblage data are available at https://doi.org/10.5281/zenodo.6948750.
R code used to generate the results of this study are available at https://doi.org/10.5281/zenodo.6948750.

Field-specific reporting

Please select the one below that is the best fit for your research. If you are not sure, read the appropriate sections before making your selection.

☐ Life sciences ☐ Behavioural & social sciences ☒ Ecological, evolutionary & environmental sciences

For a reference copy of the document with all sections, see nature.com/documents/nr-reporting-summary-flat.pdf

Ecological, evolutionary & environmental sciences study design

All studies must disclose on these points even when the disclosure is negative.

Study description	We analyse community change in fossil planktonic foraminifera assemblage data (relative abundances) using commonly used dissimilarity metrics and principal component analysis to assess how biodiversity turnover responded to past climate changes.
Research sample	We used published fossil assemblage data (see Extended Data Table 1) and one data set that is unpublished but used with the authors permission (108-658C).
Sampling strategy	The 25 records studied here were selected from 198 records situated in the North Atlantic Ocean and adjacent seas initially identified in public databases as containing planktonic foraminifera assemblage counts within the past 24 ka.
Data collection	We used previously published data that were initially identified in public databases and met our data inclusion criteria. Detailed information on the individual records and meta data can be found in Extended Data Table 1.
Timing and spatial scale	We used previously published data. The fossil assemblage data are from records situated in the North Atlantic Ocean and adjacent seas and cover the time period of 23 ka to 3 ka with an average resolution of 0.06 ka, ranging from 0.04 to 1.31 ka.<br>For the 9 sites included in the PALMOD 130k marine palaeoclimate data synthesis V1.1 (Jonkers et al., 2020), we used their provided revised age models; for the remaining 16 sites we adapted the same approach as in Jonkers et al. (2020). Further information on the age models are given in the Material and Methods sections and in Extended Data Table 1.
Data exclusions	Criteria for inclusion of records in our compilation: 1) situated in the North Atlantic Ocean and adjacent seas; 2) complete taxonomic resolution; 3) covering the entire time period of interest spanning the transition from the last ice age to the current warm period (i.e. at least 23 ka to at least 3 ka) and 4) resolution below 1.5 ka to resolve millennial-scale climate events.
Reproducibility	no experiments conducted
Randomization	no experiments conducted
Blinding	no experiments conducted

Did the study involve field work? ☐ Yes ☒ No

Reporting for specific materials, systems and methods

We require information from authors about some types of materials, experimental systems and methods used in many studies. Here, indicate whether each material, system or method listed is relevant to your study. If you are not sure if a list item applies to your research, read the appropriate section before selecting a response.

Materials & experimental systems

n/a	Involved in the study
☒	☐ Antibodies
☒	☐ Eukaryotic cell lines
☒	☐ Palaeontology and archaeology
☒	☐ Animals and other organisms
☒	☐ Human research participants
☒	☐ Clinical data
☒	☐ Dual use research of concern

Methods

n/a	Involved in the study
☒	☐ ChIP-seq
☒	☐ Flow cytometry
☒	☐ MRI-based neuroimaging
